# Influenza Outbreak Control in Confined Settings

**DOI:** 10.3201/eid1104.040845

**Published:** 2005-04

**Authors:** Ran D. Balicer, Michael Huerta, Yuval Levy, Nadav Davidovitch, Itamar Grotto

**Affiliations:** * Israel Defense Forces Medical Corps, Tel-Hashomer, Israel;; † Ben-Gurion University, Beer Sheva, Israel

**Keywords:** influenza, prevention and control, disease outbreak, mass immunization, research

## Abstract

Influenza may rapidly disseminate within populations living in confined settings, causing considerable morbidity and disrupting daily activities. We describe an influenza A outbreak on a military base where 3,000 young adults, most of whom were unvaccinated, lived in close daily contact. Visits to the base clinic by 48 persons with acute respiratory illness within 2 days allowed early identification of this outbreak and prompted immediate epidemiologic investigation. Overall, 85 personnel (2.83% of base population) met the case definition for influenzalike illness. On-site laboratory confirmation with field detection kits, rapid implementation of respiratory illness control protocols, and a mass vaccination campaign were applied to limit disease dissemination. The outbreak was halted 14 days after the mass vaccination campaign was completed. We review the control measures available for controlling influenza outbreaks in confined settings and discuss the role of rapid mass vaccination within this context.

## Abstract 2

Early reporting, on-site laboratory testing, and rapid mass vaccination may limit influenza outbreaks in confined settings.

Influenza causes substantial illness and loss of work days among young adults, and outbreaks can affect the preparedness of military units (1). The generally recommended measures for controlling influenza outbreaks (e.g., isolation, quarantine, hygiene enhancement) (2) may not be sufficient to contain an outbreak in such confined settings, when attack rate may be as high as 45% (3). We describe an influenza outbreak in which rapid identification of the causative agent permitted mass vaccination to be used as a control measure, examine the effects of this intervention on disease dissemination, and discuss the potential role of this control measure in containing influenza epidemics.

## Methods

Over a 2-day period during January 2002, 48 patients sought treatment at the clinic of a large military base in central Israel; their symptoms included fever, cough, and sore throat. This unusually high patient load prompted the clinic commander to notify headquarters, and a team from the Epidemiology Section of the Israel Defense Forces (IDF) Medical Corps arrived at the scene by the next morning (outbreak day 3) and initiated an epidemiologic investigation. The investigation team retrieved all patient visit records for the preceding 2 weeks from the clinic's computerized patient files and continued daily follow-up for 4 additional weeks. The case definitions defined acute respiratory illness (ARI) as cough, sore throat, or coryza. Influenzalike illness (ILI), a subset of ARI, was defined as ARI with temperature >37.8°C (>100.0°F). Cases were classified according to these case definitions, and epidemic curves were constructed. Demographic information, including sex, rank, unit, and influenza vaccination status, were retrieved from personnel records. Active surveillance for ARI was initiated among all post personnel.

Oropharyngeal and nasopharyngeal specimens were tested in the field by using rapid influenza tests (Influenza A/B Rapid Test, Roche Diagnostics, Basel, Switzerland), and these results were verified with immunofluorescence staining and viral culture. The IDF health corps at that time had >2,000 available doses of influenza vaccine. This vaccine was a subunit influenza preparation, containing influenza A and B strains equivalent to A/New Caledonia/20/99 (H1N1)-like, A/Moscow/10/99 (H3N2)-like, and B/Sichuan/379/99-like strains, in accordance with World Health Organizations recommendations for the Northern Hemisphere 2001–2002 winter season (4). Active surveillance was continued for 24 days to assess the effect of the employed control measures on the dissemination pattern of the outbreak.

## Results

The base housed ≈3,000 men and women, of whom 136 (4.5%) had been vaccinated against influenza during the preceding 3 months as part of routine, seasonal preventive health measures. These 136 vaccinees made up 87.7% of the vaccine-eligible personnel in this base; 19 other personnel refused to receive the vaccine that winter. Initial investigation showed that ARI incidence rates during the 2-day period January 13–14, 2002 (outbreak days 1 and 2), were indeed significantly higher than the preceding daily average rates (24 vs. 4.1 patient visits/24 h, p < 0.001). During these 2 days, ILI was responsible for a significantly higher proportion of all clinic visits, compared with visits during the preceding 2 weeks (21% vs. 4.3%, p < 0.001).

The epidemic curves of ARI and ILI are shown in the Figure. During outbreak days 1 and 2, a total of 48 cases of ARI were recorded; 34 met the criteria for ILI. None of the patients had been vaccinated against influenza in the preceding 12 months. Cases were evenly distributed across all areas of the base and among all ranks, with no specific unit on post showing an exceptionally high attack rate. On weekends the base clinic treated emergency cases only, which explains the artifactual cyclic lacunae visible on the curve.

**Figure:**
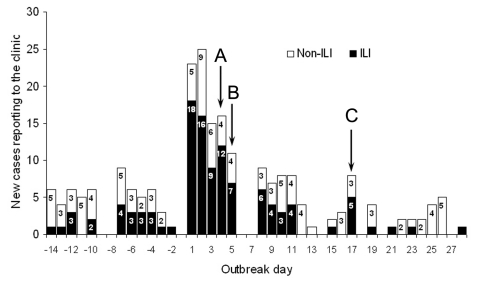
Daily incidence of acute respiratory illness (ARI) cases at base infirmary, by case definition. Shaded segment of bar indicates ARI cases that met the definition of influenzalike illness (ILI) (temperature >37.8°C). Nonshaded segment indicates ARI cases that did not meet the ILI criterion (non-ILI). Arrows: A) vaccination campaign initiation, B) vaccination campaign termination, C) day 14 after campaign initiation.

Ten oropharyngeal and nasopharyngeal specimens were tested on outbreak day 3 by using a rapid influenza kit; 1 was positive. Seven of these specimens were later found to be positive for influenza A virus by immunofluorescence staining and culture. This information was only available after the decision to undertake a mass vaccination campaign had been made and the campaign had been carried out. Typing the virus from culture showed that 6 of the 7 influenza-positive cultures were A/Moscow/10/99(H3N2)-like, a strain included in the 2001–2002 vaccine composition. Specific typing of the seventh culture was unavailable.

Control measures immediately employed from outbreak day 3 and afterward included active case finding, strict isolation, and placing patients on sick leave, and infirmary records confirm that 82.4% of patients with physician-confirmed ILI were indeed sent for sick leave off post. Despite the introduction of the above measures, new ILI patients continued to appear, as shown in the Figure. Because of the potential for further dissemination of the disease among unvaccinated personnel and the operational implications of such disease spread on troop preparedness, additional steps directed towards active intervention were initiated. As sufficient quantities of antiviral drugs were not readily available at that time, >2,200 doses of influenza vaccine were rapidly transported on outbreak day 4 to the base for use in an immediate mass vaccination campaign. Within 2 days (outbreak days 4–5), 2,118 soldiers (>70% of the base population) were vaccinated.

The outbreak had a substantial impact on military activities. Key commanders and personnel were incapacitated by their illness for several days, thus disrupting the operational routine. Entry to and exit from the base were denied, except to those with documents showing they had been vaccinated, and training operations were suspended during the outbreak in an attempt to avoid noncrucial congregation and limit disease dissemination.

Over the next 2 weeks (outbreak days 4–17), patient visits to the clinic due to ARI and ILI remained high, despite the intervention measures taken; a mean daily rate of 3 cases of ILI was maintained. As shown in the Figure, illness remained high for 17 days, after which time illness rates returned to preepidemic levels. We defined outbreak termination as the day from which the daily onset of new ILI cases was ≤4 (the highest single daily ILI incidence observed during the preoutbreak period). Although the outbreak initially appeared to have ended on outbreak day 12, the 5 ILI patients seen in the cluster on outbreak day 17 exceeded this cutoff value so that the day of termination was set at outbreak day 18. After day 18, incidence declined to a mean daily rate of 1.9 cases for ARI and 0.4 for ILI. These rates were maintained for an additional 10 days, at which time active surveillance was stopped. Overall, 140 ARI cases were recorded within 17 days, 85 of which (60.7%) met the case definition for ILI. The ARI attack rate during this outbreak was 46.7/1,000, and the ILI attack rate was 28.3/1,000. None of the patients required hospitalization.

## Discussion

An outbreak of influenza in a military base with a largely unvaccinated population was rapidly identified and was subsequently halted 14 days after a rapid mass vaccination program. Disease dissemination during influenza outbreaks in military bases can have reach attack rates as high as 37%–45% (3,5), and similar rates were noted during outbreaks on naval ships (6). With no control group in this case, we cannot predict the exact attack rate that would have been reached, had this unusual intervention not been applied. However, several factors in the setting of this outbreak lead us to believe the expected attack rate would have been much higher than 2.83%. These factors include the initial high incidence rate; the even distribution of cases across all areas of the base and among all ranks; the large substrate for disease dissemination in a base of ≈3,000 young men, mostly unvaccinated, living in confined settings and in close everyday contact; and the continuous incidence of new cases, despite conservative measures employed, an incidence that had not completely subsided until 14 days after the mass vaccination campaign. Such a low attack rate probably cannot be attributed solely to the natural course of the outbreak and seems to indicate that control measures had a substantial beneficial effect.

Although this group of healthy, working adults is not at increased risk for serious complications, influenza is not a trivial illness in this group. Prominent manifestations include increased work absenteeism, impaired work productivity when ill, and a low yet clinically important incidence of serious complications (7,8). In the military, work absenteeism may hinder units' preparedness. In the case presented, the outbreak was contained in time to prevent the base strategic abilities from being compromised.

Seasonal vaccination of young adults, mainly in crowded settings, is both highly successful and cost-effective (9,10). Routine vaccination of similar population groups, including students and other persons in institutional settings, is currently encouraged by the Advisory Committee on Immunization Practices (ACIP) recommendations for prevention and control of influenza (2). IDF conducts an annual influenza vaccination campaign, directed at crucial fighting-unit personnel and personnel with chronic respiratory and cardiac disease. Most of the personnel of the base in question (>95%) did not fall under any of these categories and were not vaccinated for influenza. Influenza vaccination is not forced during the IDF annual vaccination campaigns, and each year a variable proportion of vaccine-eligible troops refuse to be vaccinated. On this base, 136 personnel, 87.7% of the base target population, were vaccinated during that year's vaccination campaign. Nineteen vaccine-eligible subjects (12.3%) refused to receive the vaccine that winter. This refusal rate is well within the expected range, according to previous years' experience (unpub. data).

The outbreak emphasizes the crucial role of continuous surveillance for respiratory disease in the military, as rapid detection is a major factor of successful intervention. The explosive pattern of this outbreak, as demonstrated by the sudden illness rate increase in the first 2 days of the outbreak, enabled rapid detection and initiation of prompt investigation. Illness caused by influenza virus is difficult to distinguish clinically from that of other respiratory pathogens on the basis of symptoms alone. Reported sensitivity of clinical definitions for ILI (defined as fever and cough) has ranged from 63% to 78%; reported specificity has ranged from 55% to 71%, respectively, when compared to diagnosis by viral culture (11,12). In this case, decisions concerning which control measures to implement, including mass vaccination, were made on outbreak day 3. The mass vaccination campaign was carried out during outbreak days 4 and 5, several days before culture-based influenza diagnosis was available. The diagnosis was therefore based on the combination of clinical signs, epidemiologic characteristics, and results of a rapid influenza identification kit. The kit is designed to detect both influenza A and B viruses (but not to distinguish between them), with a reported sensitivity of 77.4% and specificity as high as 93% (13). These characteristics render the kit inappropriate for the diagnosis of influenza in individual patients, but the kit remains a useful tool for implicating influenza as the causative agent in large outbreaks, even if only a few patients test positive (14).

Confirmation of influenza A with immunofluorescence staining and viral culture requires at least several days, a timeframe in which an influenza outbreak may grow out of control. Decision-making regarding control measures should not necessarily be delayed until such confirmation is achieved. In this case, decisions on intervention measures were made several days before culture results became available, and the diagnosis was based on clinical and epidemiologic characteristics and the results of the rapid influenza detection kit. Guidelines for outbreak control in young adults within confined settings (i.e., military bases, correctional facilities, or college dormitories) are scarce are probably similar to guidelines used for quelling nursing home outbreaks. In the latter case, recommended measures include active identification of patients with confirmed or suspected influenza, restriction of staff movement between wards or buildings, restriction of contact between ill staff or visitors and patients, influenza vaccination for staff and patients, and use of antiviral drugs (2). Some of these recommendations may not be applicable in an active military unit, most notably isolation and widespread restriction of movement. Antiviral agents in sufficient quantities may not always be available, as in our case, but when they are available, they may play an important role in stopping such outbreaks.

Mass vaccination as a means of controlling outbreaks has several limitations. The vaccine may reach its full potential effectiveness (70%–90% reduction in influenza illness) only when the vaccine and circulating viruses are antigenically similar (2). The incubation period of the influenza virus is 1–3 days, whereas the development of antibodies in adults after vaccination can take up to 2 weeks, depending on prior vaccination and sensitization. In this time, the virus can complete several infection cycles, therefore rendering mass vaccination inappropriate as a sole measure of intervention and only appropriate when the susceptible population size is large enough that the outbreak can be expected not to subside within 2 weeks. In instances when an influenza outbreak is not contained by using routine protocol and a large portion of the population is unvaccinated and remains at risk, mass vaccination can ensure the termination of the outbreak within ≈2 weeks. When available, prophylaxis with antiviral drugs must be considered an adjunct to other control measures such as isolation of patients, hygiene enhancement campaigns, and reduction of crowding to a necessary minimum in limiting disease dissemination to a minimum during this time frame. These control measures may confer important information for unit commanders, since the 2-week period provides a point of reference for projecting troop readiness. When the population at risk carries crucial deployment responsibilities, as was the case in our outbreak, mass vaccination may be imperative.

Following this outbreak, efforts have been directed at increasing the acceptance of seasonal influenza vaccination in the specific populations within the IDF. Brochures and lectures were used to deliver evidence-based information about the benefits of influenza vaccination to both the troops and the base physicians. Assessment of the effect of these measures on compliance with influenza vaccination is now underway.

When feasible and affordable, preventive seasonal influenza vaccination is preferable to rapid vaccination during an outbreak because of the above-described caveats of the latter strategy. For the time being, however, the IDF will continue to focus its annual influenza vaccination campaigns on specific groups, mainly because of the high costs associated with universal coverage. Under these circumstances, influenza outbreaks such as the one presented here can be expected to recur, and adequate quantities of antiviral agents and vaccines must be made readily available to control future outbreaks. Active surveillance for ILI, including continuous laboratory sampling, is now under way in specific field units within the IDF in an attempt to uncover the viral pathogens that account for ILI and estimate the true incidence of influenza in unvaccinated subpopulations. Since recent outbreaks of highly pathogenic avian influenza in East Asia may herald the next influenza pandemic, heed must be taken now to implement and evaluate an array of outbreak control measures during the interpandemic period.

## Conclusions

Early reporting of a potential influenza outbreak among soldiers, on-site laboratory confirmation with field detection kits, and rapid implementation of mass vaccination combined with a respiratory illness control protocol likely limited the magnitude of this outbreak. In confined settings, when the threat of an acceleration of the outbreak is substantial, antiviral drugs are not available in abundant amounts, and sufficient vaccine doses are available, mass vaccination of the population at risk should be considered because it should ensure the termination of the outbreak within 10–14 days. Conservative control measures must serve as adjuncts to limit disease dissemination during this period until the vaccine takes effect.

## References

[1] Grotto I, Mandel Y, Green MS, Varsano N, Gdalevich M, Ashkenazi I, Influenza vaccine efficacy in young, healthy adults. Clin Infect Dis. 1998;26:913–7. 10.1086/513934 9564475

[2] Centers for Disease Control and Prevention. Prevention and control of influenza: recommendations of the Advisory Committee on Immunization Practices (ACIP). MMWR Recomm Rep. 2004;53:1–40. 15163927

[3] Makras P, Alexiou-Daniel S, Antoniadis A, Hatzigeorgiou D. Outbreak of meningococcal disease after an influenza B epidemic at a Hellenic Air Force Recruit Training Center. Clin Infect Dis. 2001;33:e48–50. 10.1086/322609 11512107

[4] World Health Organization. Recommended composition of influenza virus vaccines for use in the 2001–2002 season. Wkly Epidemiol Rec. 2001;76:58–61. 11236648

[5] Klontz KC, Hynes NA, Gunn RA, Wilder MH, Harmon MW, Kendal AP. An outbreak of influenza A/Taiwan/1/86 (H1N1) infections at a naval base and its association with airplane travel. Am J Epidemiol. 1989;129:341–8. 2912044 10.1093/oxfordjournals.aje.a115137

[6] Earhart KC, Beadle C, Miller LK, Pruss MW, Gray GC, Ledbetter EK, Outbreak of influenza in highly vaccinated crew of U.S. Navy ship. Emerg Infect Dis. 2001;7:463–5. 11384530 10.3201/eid0703.010320 PMC2631796

[7] Barker WH, Mullooly JP. Impact of epidemic type A influenza in a defined adult population. Am J Epidemiol. 1980;112:798–811. 7457471 10.1093/oxfordjournals.aje.a113052

[8] Ahmed F, Singleton JA, Franks AL. Clinical practice. Influenza vaccination for healthy young adults. N Engl J Med. 2001;345:1543–7. 10.1056/NEJMcp011924 11794222

[9] Nichol KL, Lind A, Margolis KL, Murdoch M, McFadden R, Hauge M, The effectiveness of vaccination against influenza in healthy, working adults. N Engl J Med. 1995;333:889–93. 10.1056/NEJM199510053331401 7666874

[10] Turner D, Wailoo A, Nicholson K, Cooper N, Sutton A, Abrams K. Systematic review and economic decision modelling for the prevention and treatment of influenza A and B. Health Technol Assess. 2003;7:1–170. 14609480 10.3310/hta7350

[11] Boivin G, Hardy I, Tellier G, Maziade J. Predicting influenza infections during epidemics with use of a clinical case definition. Clin Infect Dis. 2000;31:1166–9. 10.1086/317425 11073747

[12] Monto AS, Gravenstein S, Elliott M, Colopy M, Schweinle J. Clinical signs and symptoms predicting influenza infection. Arch Intern Med. 2000;160:3243–7. 10.1001/archinte.160.21.3243 11088084

[13] Thomas Y, Kaiser L, Wunderli W; EISS Task Group on Near Patient Test. The use of near patient tests in influenza surveillance: Swiss experience and EISS recommendations. Euro Surveill. 2003;8:240–6. 14724333

[14] Wunderli W, Thomas Y, Muller DA, Dick M, Kaiser L. Rapid antigen testing for the surveillance of influenza epidemics. Clin Microbiol Infect. 2003;9:295–300. 10.1046/j.1469-0691.2003.00650.x 12667239

